# The Marine Food Chain in Relation to Biodiversity

**DOI:** 10.1100/tsw.2001.85

**Published:** 2001-10-19

**Authors:** Andrew R.G. Price

**Affiliations:** Ecology and Epidemiology Group, Department of Biological Sciences, University of Warwick, Coventry CV4 7AL, UK

**Keywords:** seafood, fishing, ecosystems, environmental impacts, governance

## Abstract

Biodiversity provides “raw materials” for the food chain and seafood production, and also influences the capacity of ecosystems to perform these and other services. Harvested marine seafood species now exceed 100 million t y -1 and provide about 6% of all protein and 17% of animal protein consumed by humans. These resources include representatives from about nine biologically diverse groups of plants and animals. Fish account for most of the world’s marine catches, of which only 40 species are taken in abundance. Highest primary productivity and the richest fisheries are found within Exclusive Economic Zones (EEZ). This narrow strip (200 nautical mile/370 km wide) is not only the site of coastal “food factories” but also the area associated with heaviest perturbation to the marine environment. Structural redundancy is evident in marine ecosystems, in that many species are interchangeable in the way they characterise assemblage composition. While there is probably functional redundancy within groups, the effects of species loss on ecosystem performance cannot be easily predicted. In particular, the degree to which biodiversity per se is needed for ecosystem services, including seafood/fishery production, is poorly understood. Many human activities, including unsustainable fishing and mariculture, lead to erosion of marine biodiversity. This can undermine the biophysical cornerstones of fisheries and have other undesirable environmental side effects. Of direct concern are “species effects”, in particular the removal of target and non-target fishery species, as well as conservationally important fauna. Equally disrupting but less immediate are “ecosystem effects”, such as fishing down the food web, following a shift from harvested species of high to low trophic level. Physical and biological disturbances from trawl nets and dynamite fishing on coral reefs can also severely impact ecosystem structure and function. “Broadscale” biological and social effects brought about by fishing carry even more far-reaching consequences. For example, fishing itself can change the age at which sexual maturity is reached, thus affecting the reproductive status of the stock. Hence, fishing may be regarded as a mediator of evolution. Social impacts include conflicts over fish prices and policies arising from heavy fishing and inadequate institutional structures. Measures to increase the sustainability of catches and of biodiversity need to be much more tightly coupled. Promising approaches include use of bio-economic indicators and fully protected marine areas. High- and local-level governance options are also examined. Use of expert systems incorporating “fuzzy logic” are providing useful environmental insights in the ASEAN countries and other parts of the world, and have applications in fishery management and biodiversity conservation.

## INTRODUCTION

Marine food chains leading to seafood species are among the many goods and services generated by marine ecosystems. Their utilisation has been a characteristic of human societies since earliest times. In the Red Sea, for example, this began during the Middle Palaeolithic[1], i.e., at least 100,000 years ago. Initially, relatively few species were harvested, and impacts on stocks and marine biodiversity would have been modest compared with the effects of fishing today. However, population failures of exploited species are not just recent phenomena. One example is green turtles in the Cayman Islands, the fishery for which crashed the 18$^{th}$ century and was gone by 1800[2]. During the same century Steller's sea cow was hunted to extinction.

The 20$^{th}$ century, in particular, has seen the greatest change. Expanding populations and markets, coupled with improved fishing technologies, have greatly increased the range of exploitable and exploited seafood species. Resource exploitation now directly targets more components of marine biodiversity than ever before. But it is also evident that marine biodiversity — the structural and functional cornerstones of marine food chains — is being eroded by a multitude of activities. Ironically, these include fishing itself and mariculture.

The overall objective of this paper is to examine associations between marine food chains, human activities that exploit them, and biodiversity. Two fundamental questions are 1) to what extent are seafood production and overall marine biodiversity of an ecosystem interdependent, and 2) how seriously are fishing and aquaculture, as human impacts, eroding biodiversity?

Following the introduction, the paper reviews the direct value of biodiversity, as "raw materials" for the food chain and seafood species harvested by humans. It also examines links between harvested species and overall biodiveristy of the marine ecosystem. This is followed by assessment of the impacts of fishing and related activities on marine biodiversity and the environment. The paper concludes with examination of wider governance implication of the marine food chain and its interconnections with biodiversity, considering both technical/scientific and legal aspects.

## THE FOOD CHAIN AS AN INTEGRAL COMPONENT OF BIODIVERSITY

### Provision of Seafood Species

Seafood and other marine species can be classified according to habitat (e.g., demersal/pelagic species) and, linked to this, fishing method (demersal trawling, drift net and purse-seining, lines, traps and cages, or explosives). Marine resources can also be classified by mode of feeding (e.g., herbivore, planktivore, or piscivore) or behaviour (e.g., anadromous or catadramous [Table 1]). Taxonomic grouping is another obvious division, and one clearly relevant to this paper.

**TABLE 1**
**World Catches (mt $\times 10^6$) of Marine Living Resources by Species Groups***

| ***Species Group*** | **1990** | **1991** | **1992** | **1993** | **1994** | **1995** | **1996** |
|---|---|---|---|---|---|---|---|
| FISH | | | | | | | |
| Flounders, halibuts, soles | 1.21 | 1.08 | 1.16 | 1.09 | 0.98 | 0.91 | 0.92 |
| Cods, hakes, haddocks | 11.58 | 10.24 | 10.43 | 9.92 | 9.64 | 10.61 | 10.71 |
| Redfishes, basses, congers | 5.73 | 5.93 | 5.96 | 5.69 | 6.31 | 6.85 | 6.61 |
| Jacks, mullets, sauries | 9.59 | 10.16 | 10.36 | 9.96 | 9.88 | 10.84 | 11.14 |
| Herrings, sardines, anchovies | 22.32 | 21.72 | 21.20 | 21.89 | 25.84 | 21.97 | 22.32 |
| Tunas, bonitos, billfishes | 4.40 | 4.59 | 4.51 | 4.55 | 4.62 | 4.71 | 4.58 |
| Mackerels, snoeks, cutlassfishes | 3.55 | 3.47 | 3.45 | 4.01 | 4.51 | 4.69 | 5.14 |
| Sharks, rays, chimaeras | 0.69 | 0.71 | 0.73 | 0.74 | 0.75 | 0.76 | 0.76 |
| Misc. marine fishes | 10.04 | 9.91 | 10.66 | 11.13 | 11.03 | 11.36 | 11.99 |
| CRUSTACEANS | | | | | | | |
| Sea spiders, crabs | 0.87 | 1.00 | 1.04 | 1.03 | 1.21 | 1.17 | 1.22 |
| Lobsters, spiny rock lobsters | 0.21 | 0.22 | 0.21 | 0.21 | 0.21 | 0.22 | 0.21 |
| Squat lobsters | <0.1 | <0.1 | <0.1 | <0.1 | <0.1 | 0.01 | 0.01 |
| Shrimps, prawns | 1.97 | 2.02 | 2.09 | 2.09 | 2.25 | 2.28 | 2.47 |
| Krill, planktonic crustaceans | 0.37 | 0.36 | 0.31 | 0.09 | 0.08 | 0.12 | 0.10 |
| Misc. marine crustaceans | 0.46 | 0.58 | 0.62 | 0.83 | 0.90 | 1.03 | 1.09 |
| MOLLUSCS | | | | | | | |
| Abalones, winkles, conches | 0.08 | 0.08 | 0.08 | 0.09 | 0.10 | 0.10 | 0.10 |
| Oysters | 0.13 | 0.11 | 0.13 | 0.13 | 0.13 | 0.17 | 0.16 |
| Mussels | 0.26 | 0.25 | 0.28 | 0.27 | 0.28 | 0.24 | 0.20 |
| Scallops, pectens | 0.53 | 0.47 | 0.51 | 0.48 | 0.60 | 0.50 | 0.46 |
| Clams, cockles, arkshells | 1.04 | 0.97 | 1.01 | 1.01 | 0.94 | 0.96 | 0.93 |
| Squids, cuttlefish, octopuses | 2.26 | 2.57 | 2.74 | 2.72 | 2.77 | 2.86 | 3.04 |
| Misc. marine molluscs | 0.66 | 0.67 | 0.90 | 1.13 | 1.22 | 1.41 | 1.05 |
| MARINE MAMMALS (no. $\times 10^3$) | | | | | | | |
| Blue whales, fin whales | 0.64 | 0.66 | 0.55 | 0.73 | 0.86 | 0.98 | 1.21 |
| Sperm whales, pilot whales | 114 | 72 | 40 | 42 | 38 | 22 | 22 |
| Eared seals, hair seals, walruses | 184 | 201 | 216 | 160 | 220 | 162 | 369 |
| Misc. aquatic mammals | 2.09 | 1.64 | 1.26 | 1.55 | 1.61 | 1.26 | 1.75 |
| REPTILES | | | | | | | |
| Turtles | <0.1 | <0.1 | <0.1 | <0.1 | <0.1 | <0.1 | <0.1 |
| ASCIDIANS | | | | | | | |
| Sea-squirts, other tunicates | <0.1 | <0.1 | <0.1 | <0.1 | <0.1 | <0.1 | 0.21 |
| MERESTOMATA | | | | | | | |
| Horseshoe crabs, other arachnoids | <0.1 | <0.1 | <0.1 | <0.1 | <0.1 | <0.1 | <0.1 |
| ECHINODERMS | | | | | | | |
| Sea urchins, other echinoderms | 0.95 | 0.10 | 0.10 | 0.11 | 0.12 | 0.13 | 0.12 |
| Misc. aquatic invertebrates | 0.15 | 0.25 | 0.49 | 0.28 | 0.31 | 0.43 | 0.52 |
| ALGAE | | | | | | | |
| Seaweed, other aquatic plant production[a] | 1.18 | 1.05 | 1.05 | 0.98 | 1.05 | 1.16 | 1.12 |

*Salmon, eels and other fish spending part of their life cycle in freshwater are not included in the table. These include anadramous fish (e.g., salmon, sturgeon, shad, smelt, sea lampreys) which breed in freshwater, whereas adults spend most of their lives at sea; and catadramous fish (e.g., American and European eels), which breed in the sea but spend the majority of adult life in freshwater.

[a]Some used for human consumption, but mostly for other products.

Data taken from Reference 56.

Harvested marine fish and other living marine resources fall into nine principal taxonomic/species groups, based on a global compilation of catch records (Table 1). These include biologically diverse representatives of plants and animals (invertebrates and vertebrates) which include: (1) algae/aquatic plants; (2) crustaceans; (3) molluscs; (4) reptiles; (5) merestomata (horseshoe crabs and other arachnoids); (6) echinoderms (sea urchins and sea cucumbers); (7) ascidians (sea squirts); (8) fish; and (9) marine mammals.

Fish are the dominant group within the marine food chain in terms of global catches (Table 1). Only forty of the world’s > 20,000 fish species[3] are taken in abundance. Many more species are taken in smaller quantities, for example by multi-species tropical artisanal fisheries. Six groups (cod, herring, jack, redfish, mackerel, and tuna) account for nearly two thirds of the annual commercial marine fish catch. This is not unlike the pattern for terrestrial food production, in which only 15 plant and 8 animal species supply 90% of the "global larder", with just four crops (wheat, rice, corn, and potato) accounting for more food production than all other crops combined[4].

More than 100 million tonnes of fish are caught from the sea annually[5]. Direct consumption of fish and shellfish worldwide provides about 6% of all protein and 17% of animal protein consumed by humans; a further 5% of human protein consumption derives indirectly from livestock fed with fish meal[6]. Of the total world population of >6 billion, an estimated 1 billion, principally from Africa and Southeast Asia, rely on fish and shellfish as their staple protein[6]. The ocean accounts for more than 80% of the annual catch, of which 99% is taken from coastal waters. These are by far the most productive parts of the ocean and include estuaries, mangroves, marshes, seagrasses, coral reefs, and areas of upwelling. Principally by the action of offshore winds, cold, nutrient-rich water comes to the surface from below to replace the warmer, nutrient-depleted water of surface waters. These coastal "food factories" lie within 370 km (200 nautical miles) of the coastal, i.e., within coastal states’ Exclusive Economic Zones (EEZ). Yet it is this same narrow coastal strip which is subject to the heaviest pressures in the marine environment[7].

Global aquaculture now accounts for 25% of fish consumption and has doubled in weight and output between 1986 and 1996[8]. Farmed shrimp alone have an annual value of more than $6 billion.

## Biodiversity and Ecosystem Integrity in Relation to the Food Chain

The extent to which fish and other seafood production depend on biodiversity of the ecosystem is a critical but poorly understood issue. Marine macrofauna (retained on 200- to 500-µm sieves) of marine sediments are the most extensive faunal assemblage on earth, playing critical functional roles in biogeochemical processes[9]. They are particularly important in secondary production, both as a direct food source (e.g., clams) and as major food sources for bottom-feeding (demersal) species that are commercially fished[10]. Many species have been shown to be interchangeable in the way they characterise assemblage composition, suggesting *structural redundancy* to be remarkably high[11].

*Functional redundancy*, however, is more complex. Certainly, complete loss (or, in some cases, addition) of particular macrofaunal species, keystone predators or a trophic group can substantially disrupt ecosystem functioning[9,12]. In San Francisco Bay, invasion of a suspension-feeding bivalve species (*Potamocorbula amurensis*) resulted in reduced water column primary production[13]. A contrasting example is the invasion of the Asiatic suspension-feeding clam, *Corbicula fluminea*, in the Potomac River Estuary (Washington, D.C.), which led to a decrease in water turbidity but increase in submerged macrophytes, bird, and fish populations[14]. While there is probably appreciable functional redundancy within groups, it is generally not possible to predict the effects of species loss on ecosystem performance[15]. Often several species seem to be occupying the same trophic role, suggesting that biodiversity loss might be inconsequential in many cases. However, it would be premature to generalise between redundancy for one specific ecological role and another and to extend this generalization to ecosystem services overall[9]. A study of seagrasses reveals strong positive association between biodiversity and marine ecosystem functions, together with the services yielded to humanity. In general, and in response to the first fundamental question raised, the degree to which biodiversity per se is needed for ecosystem services[9,12,15] and seafood production is still unclear. For example, following an ecosystem perturbation, fish harvests might decline directly from the impact(s), or indirectly from biodiversity reduction caused by the impact(s). A further issue is that biodiversity is a multi-faceted concept and measure[16,17], and a particular impact can even result in an increase in biodiversity, according to some measures (Shannon Wiener, Simpson's index), yet a decrease, according to others (taxonomic distinctness[18]). Because of the many uncertainties, a precautionary approach is advocated, with efforts directed at minimizing biodiversity loss from fishing, pollution and habitat degradation, as well as global warming[15].

## IMPACTS OF FOOD CHAIN HARVESTING ON BIODIVERSITY

### Fishing

Many components of biodiversity contribute to global catches of seafood and other living marine resources, as shown above. Some fisheries appear to be sustainable, such as the *Loligo* squid fishery in Falklands waters[19], but many are not. This section shows that fishing itself can lead to erosion of biodiversity, undermine biophysical cornerstones of fisheries and have other undesirable environmental side effects (Table 2). Fishery species face particularly acute problems when critical life-cycle phases are disrupted by heavy fishing and/or other environmental pressures, particularly in spawning areas and nursery areas[20,21,22].

Capture fisheries are currently in global crisis and have created many problems. In place of renewable stocks for society's future use, many of the world's fisheries have left behind a litany of disasters. These range from stock collapses, reduction of higher order animals such as cetaceans[23], environmental impacts, and unsustainability to social conflict, dislocation, and economic downturns.

Reduced stock size of target fishery species is the effect of fishing generally acknowledged and best understood. Less obvious, indirect impacts have also affected biodiversity. These include increased mortality of non-target species, such as trash fish, and conservationally important species captured inadvertently as by-catch[23]. These impacts are referred to in Table 2 as "Species Effects". "Ecosystem effects" of fishing go far beyond just species and are equally problematic. Acute examples include extensive biophysical damage to coral reefs and the seabed caused by trawl nets and dynamite fishing on coral reefs. A more subtle but no less significant disturbance is "fishing down marine food webs"[24]. This results in loss of higher trophic-level species from fishing, causing destabilization of food webs and eventually leading to loss of productivity. However, other factors may also be at play, such as altered harvesting patterns (e.g., of short-lived invertebrates later in the time series), which is consistent with a move downwards in trophic-level targeting[25].

"Broadscale" biological and social effects brought about by fishing carry even more far-reaching consequences (Table 2). For example, fishing itself can change the age at which sexual maturity is reached, thus affecting the reproductive status of the stock. Hence, fishing may be regarded as a mediator of evolution. Further details of fisheries effects on ecosystems and biodiversity are given in recent reviews[26,27]. Economic and sociopolitical effects of fishing are now global in extent, highly complex, and not always predictable. These are seldom adequately integrated with biological and technical aspects of fisheries[28,29,30].

**TABLE 2**
**Direct and Indirect Effects of Fishing on Species, Ecosystems, and Biodiversity**

| Fishing Effect | Impact(s) or Consequence(s) | References |
|---|---|---|
| SPECIES EFFECTS | | |
| Removal of target fish species | >70% of world's commercially important marine fish stocks fully fished, over-exploited, depleted, or slowly recovering; increased likelihood of species loss or extinction caused by overexploitation | 42, 43, 44 |
| By-catch (1): removal of non-target fish species (e.g., under-sized marketable species, species not currently marketable, or target species) | By-catch, or discards, range from 17.9 to 39.5 million t $y^{-1}$ or an average of 27 million t $y^{-1}$; this replacement of high value fish by "trash" fish represents enormous wastage of fishery resources | 45, 46 |
| By-catch (2): removal of conservationally important fauna (e.g., marine mammals, turtles, and birds) | Capture of porpoises in gill nets and of turtles in trawl nets not fitted with turtle exclusion devices (TEDI); incidental mortality of species long-lived and with low reproductive rates (*K*-selected species) is a major conservation problem | 47 |
| ECOSYSTEM EFFECTS | | |
| Fishing down marine food webs | Stagnating or declining catches, i.e., unsustainability of fisheries, following shift from landings of high trophic-level, long-lived species (*K*-selected species) to low trophic-level, short-lived species (*r*-selected species) | 24 |
| Food web competition | 25-34% of primary production in shelf waters (which provide 95% of catches) is harvested, bringing humanity into competition for primary productivity with other apex predators (e.g., seabirds, marine mammals) | 37, 48 |
| Other biological effects on ecosystem structure | Reduced species diversity and local extinctions of both fishery and other e.g., keystone species; phase shifts from removal of fish predators of urchins, reducing reef accretion, and possibly increasing erosion; interactions between fishing and other agents to impair ecosystem recovery following hurricanes and other natural disasters | 27, 48, 49 |
| Physical effects on ecosystems | Physical (and biological) ecosystem disturbances from trawl nets and dynamite fishing | 50, 51, 52 |
| BROADSCALE EFFECTS | | |
| Evolutionary effects | Trend toward removal of larger sized fish, slower growth, and decline in age and/or size at sexual maturity; changes result from compensatory responses as well as genetic effects | 53, 54 |
| Genetic effects | Some evidence of reduced genetic diversity, e.g., orange roughy, virgin stocks of which have been harvested beyond their maximum sustainable yield | 27, 55 |
| Economic and political effects | Conflicts over fish prices, financial insecurity, and political dislocation, arising from combination of heavy fishing of shared stocks and inadequate institutional management structures | 29 |

## Mariculture

Like fishing, aquaculture often results in negative environmental impacts[8], including the direct or indirect loss of biodiversity. Common problems include: chemical and biological pollution (e.g., nutrients) from shrimp and other farms; misuse of chemicals, such as antibiotics; introduction of exotic species and diseases, including passing through food chain of *Escherichia coli*, Norwalk-type viral diseases, shigellosis, and cholera, as well as their spread from one ecosystem to another[31,32]; degradation of mangroves and other natural ecosystems; abandonment of aquaculture ponds, which can then be unsuitable for other activities, such as agriculture; and use of large amounts of fish oil and fishmeal taken from wild caught fish for farmed carnivorous species. These and other problems have been addressed in a recent review[8]. Insuffiently prominent in many reviews and critiques, however, is the fact that there are now many examples of good (sustainable) aquaculture practice[33,34].

Hence, in response to the second fundamental questions raised, it is evident that mariculture and fisheries can be sustainable. However, there are many instances of both practices eroding biodiversity and undermining ecosystem functions, aside from the obvious effects of stock depletion by capture fisheries.

## WIDER GOVERNANCE IMPLICATIONS

Measures to increase the sustainability of catches and of biodiversity needs to be much more tightly coupled. One approach is assessment of fishery performance through use of bio-economic indicators, which requires recognition of the uncertainty of marine systems[35]. There is also compelling evidence that use of fully protected marine areas can optimise fishery yields and, at the same time, conserve biodiversity in the face of uncertainty[36,37].

One current, high-level initiative is examining possible cooperation between Regional Seas conventions of the United Nations Environment Programme (UNEP) and regional fishery bodies in the field of ecosystem-based management of fisheries[38]. This should be beneficial to both fisheries and biodiversity. At the local level, customary marine approaches can act in a similar way, despite dilution by western influence during recent decades[39]. In the Philippines, the value of mangroves to fisheries and coastal conservation has been long recognised. Problems arose in the 1980s, however, when local people could obtain coastal tenure only if mangroves were cleared for shrimp mariculture sites. This was done, to satisfy government policy requirements but against their better judgement, leading to an array of problems for fisheries, mariculture, and coastal biodiversity. Another critical issue which needs to be addressed further, in view of the considerable local and global consequences, is the extent to which mariculture products should be for "fish or finance".

Use of expert systems incorporating "fuzzy logic" are providing useful environmental insights in the ASEAN countries and other parts of the world[40,41,42], and have applications in fishery management and biodiversity conservation. Fuzzy logic operates using a system of generic rules that involves trade-offs between data resolution and computing speed and also has other benefits.

## ACKNOWLEDGEMENTS

The suggestions of J. McGlade, C. Roberts, and C. Sheppard are acknowledged with thanks.

This paper is based on input to project, "Biodiversity: Its Importance to Human Health" supported by Center for Health and the Global Environment, Harvard University, the World Health Organization, and the UN Environment Programme. This and other documents will form the basis of a report to be published and presented at a Special Session of the UN General Assembly, June 2002. Its ultimate aim is to provide input to UN Convention on Biological Diversity and to the major international effort, "The Millennium Ecosystem Assessment".
